# 

**Published:** 1868-09-15

**Authors:** 


VOL. XXV. NUMBER 18.

### THE CHICAGO MEDICAL JOURNAL.

*PUBLISHED SEMI-MONTHLY.*

J. ADAMS ALLEN, M.D., LL.D.
EDITOR.
WALTER HAY, A.M., M.D.
ASSOCIATE EDITOR.

SEPTEMBER 15, 1868.

CHICAGO:
CHURCH, GOODMAN AND DONNELLEY, STEAM BOOK AND JOB PRINTERS,
108 AND 110 DEARBORN STREET.

